# The Potential of Silver Diamine Fluoride in Non-Operative Management of Dental Caries in Primary Teeth: A Systematic Review

**DOI:** 10.3390/medicina60111738

**Published:** 2024-10-23

**Authors:** Kornelija Rogalnikovaitė, Julija Narbutaitė, Vilija Andruškevičienė, Eglė Aida Bendoraitienė, Jaunė Razmienė

**Affiliations:** Department of Preventive and Paediatric Dentistry, Faculty of Odontology, Medical Academy, Lithuanian University of Health Sciences, J. Lukšos-Daumanto Str. 6, LT-50106 Kaunas, Lithuania; julija.narbutaite@lsmu.lt (J.N.); vilija.andruskeviciene@lsmu.lt (V.A.); egleaida.bendoraitiene@lsmu.lt (E.A.B.); jaune.razmiene@lsmu.lt (J.R.)

**Keywords:** dental caries, primary teeth, silver diamine fluoride, cariostatic agents

## Abstract

*Background and Objectives:* Dental caries has seen an increase in untreated cases, leading to significant health and quality-of-life impacts, necessitating innovative approaches like the promising non-operative management with silver diamine fluoride. This study aimed to evaluate the mechanisms of action of silver diamine fluoride on arresting dental caries in primary teeth. *Materials and Methods:* A systematic search was conducted across MEDLINE (PubMed), Google Scholar, and Wiley Online Library, including both in vivo and in vitro studies published from 1 January 2017 to 16 October 2022. The Cochrane Risk of Bias Tool assessed bias in in vivo studies, while the Quality Assessment Tool for In Vitro Studies evaluated the methodological quality of in vitro studies. *Results:* Nineteen publications met the inclusion criteria. Two studies indicated that silver diamine fluoride application significantly alters oral microflora, contributing to caries arrest. Additionally, two studies reported increased mineral density and mineral content in demineralised primary teeth, emphasising silver diamine fluoride’s role in promoting remineralisation. Three studies demonstrated significant improvements in surface microhardness, enhancing tooth resistance. However, no significant qualitative changes in bacterial species composition were noted. Modified silver diamine fluoride application techniques, including light curing or laser irradiation, enhanced efficacy, with light curing notably increasing surface microhardness. Based on a limited number of studies, no statistically significant differences in clinical effectiveness were observed with higher silver diamine fluoride concentrations or extended application durations. *Conclusions*: Silver diamine fluoride effectively induces quantitative changes in oral microflora and enhances the microhardness and mineral density of enamel and dentine in primary teeth, with modified application methods showing potential for improved outcomes.

## 1. Introduction

Dental caries, a complex condition influenced by multiple factors, remains a prevalent chronic disease among children [1]. Despite ongoing efforts to implement preventive measures and modify behavioural factors, high rates of dental caries persist [2,3]. Between 1990 and 2019, the prevalence of dental caries in primary dentition decreased by an average of 3% [4], but new cases rose by 6%, leading to a staggering 1.15 billion untreated primary dental caries cases [5]. More than 75% of untreated caries in deciduous teeth occur in middle-income countries, where healthcare systems and resources are often insufficient to address the issue. Among WHO regions, the Western Pacific region has the highest prevalence of primary teeth caries at 46%, while the African region has the lowest at 39%. The Southeast Asia region has the highest number of cases, estimated at 135 million, whereas the European region has the lowest, with approximately 41 million cases [6]. Overall, Uribe et al. [7] found that nearly 48% of children under four years old have early childhood caries, highlighting the significant burden of caries in preschool children globally.

Untreated dental caries can lead to various complications, including odontogenic infections, disrupted sleep patterns, and nutritional disorders, all of which significantly impact a child’s quality of life. Research has also established links between untreated dental caries and academic absences, attention difficulties, limited socialisation, and toothaches [8,9]. It is essential to note that treating caries in primary teeth is technically challenging and depends on factors such as the extent of damage, the child’s age, behaviour, cooperation, and financial considerations. This treatment can be costly, rendering healthcare services inaccessible to certain individuals [10]. Even with available treatment services, the management of dental caries in primary teeth frequently requires interventions performed under general anaesthesia to alleviate pain and anxiety [11]. On the other hand, it was found that treatment under general anaesthesia is associated with compromised oral health and an increased likelihood of dental fear and anxiety in the future. Additionally, children whose primary teeth were treated under general anaesthesia exhibited a higher severity of caries in their permanent dentition [12]. Consequently, considerable attention has been directed towards innovative, advanced methods for caries prevention, control, and treatment.

Non-operative management of dental caries presents a promising alternative to traditional invasive treatment methods. This approach focuses on preserving the natural tooth structure by restoring its biological and physical properties through various remineralisation techniques. Examples of these methods include the use of sodium fluoride (NaF), calcium phosphate, amorphous calcium phosphate, casein phosphopeptide-amorphous calcium phosphate, zinc-substituted hydroxyapatite, bioactive glass, self-assembling peptide (P11-4), dental paste containing appropriate fluoride levels, and more [13,14]. Silver diamine fluoride (SDF) has gained widespread acceptance for treating asymptomatic primary teeth affected by caries [11]. Among the various remineralising agents used in dentistry, SDF stands out due to its exceptionally high fluoride ion concentration (44,800 ppm), enabling the effective arrest of the caries process and the prevention of new lesion formation [15]. While NaF has been considered the ‘gold standard’ for conservative caries management for over five decades, its efficacy in arresting dentine caries has been found wanting [16,17]. Recent studies have demonstrated that SDF can arrest approximately 80% of caries lesions [18,19], significantly outperforming NaF, which arrests only about 37% [20]. High quality evidence suggests that the arrestment of caries at 12 months promoted by SDF is 66% higher than by other active material [21]. SDF is also known to inhibit the metabolic activity of cariogenic bacteria. Ishiguro et al. [22] showed that SDF caused smaller changes in pH levels compared to NaF, and the released silver and fluoride ions significantly inhibited carbohydrate fermentation. These findings suggest that SDF could be a superior alternative to traditional fluoride therapy.

SDF is believed to reduce tooth sensitivity and arrest the progression of caries lesions due to the synergistic effect of high concentrations of silver and fluoride ions, which exhibit bactericidal properties against the oral microflora involved in caries development. SDF also promotes remineralisation and inhibits the breakdown of dentine collagen fibres [23]. There is increasing evidence of the effectiveness, safety and ease of use of SDF [24,25,26,27,28]. However, ongoing scientific debates surround the precise mechanisms by which this material exerts its therapeutic effects, as the exact molecular pathways and interactions involved are not fully understood. Therefore, the aim of this study was to conduct a comprehensive evaluation and discussion of the mechanisms that contribute to the caries-arresting properties of SDF in primary teeth.

## 2. Materials and Methods

### 2.1. Protocol and Registration

The Preferred Reporting Items for Systematic Reviews and Meta-Analyses (PRISMA) [29] guidelines were followed by conducting a systematic literature search, clearly defining inclusion and exclusion criteria, and performing rigorous data extraction. The review was registered on the PROSPERO system under the registration number CRD42023432033 (https://www.crd.york.ac.uk/prospero/display_record.php?RecordID=432033, accessed on 14 June 2023).

### 2.2. Focus Question

The following focus question was developed using the PICO (Population, Intervention, Comparison, Outcome) study design, as outlined in Table 1: does treatment with SDF exhibit antibacterial and remineralising effects?

### 2.3. Information Sources and Search

The literature search was indeed conducted by two independent researchers, K.R. and J.R., and performed in MEDLINE (PubMed), Google Scholar, and Wiley Online Library electronic databases. The search was initiated on 11 September 2022, and the last search was performed on 16 October 2022. The following keywords and their combinations were used: (“silver diamine fluoride” OR “silver diammine fluoride”) AND (“dental plaque” OR (“dental” AND “plaque”) OR “oral biofilm” OR (“oral” AND “biofilm”) OR “antibacterial activity” OR (“antibacterial” AND “activity”) OR “antibacterial effect” OR (“antibacterial” AND “effect”) OR “antibacterial potential” OR (“antibacterial” AND “potential”) OR “antibacterial efficacy” OR (“antibacterial” AND “efficacy”) OR “antimicrobial effect” OR (“antimicrobial” AND “effect”) OR “antimicrobial activity” OR (“antimicrobial” AND “activity”) OR “antimicrobial potential” OR (“antimicrobial” AND “potential”) OR “antimicrobial efficacy” OR (“antimicrobial” AND “efficacy”) OR “microbiota”). Additionally, a separate search was performed to identify publications specifically focused on the remineralisation potential of SDF: (“silver diamine fluoride” OR “silver diammine fluoride”) AND (“dentin remineralisation” OR (“dentin” AND “remineralisation”) OR “enamel remineralisation” OR (“enamel” AND “remineralisation”) OR “microhardness” OR “dentin demineralisation” OR (“dentin” AND “demineralisation”) OR “enamel demineralisation” OR (“enamel” AND “demineralisation”)). The literature search primarily targeted publications from the past five years, and filters were applied to the databases to include recent articles. A comprehensive summary of the search results for articles obtained from these electronic databases can be found in Table S1 (Supplementary Materials).

### 2.4. Selection of Studies

The identified publications underwent a rigorous review process conducted by two independent reviewers, K.R. and J.R. This review process encompassed several stages. Firstly, duplicate articles were identified and subsequently removed from the initial list of publications. Subsequently, the relevance of these articles was assessed by reviewing their titles and abstracts. Lastly, the complete text versions of the selected articles were meticulously examined, following predetermined inclusion and exclusion criteria. The inter-examiner Kappa score (Cohen kappa coefficient) was 0.89 for study selection. In case of any disagreements regarding the inclusion of specific articles in the systematic review, resolution was achieved through consultation with an additional independent investigator, J.N.

### 2.5. Inclusion and Exclusion Criteria

The criteria used for inclusion during the article selection process were as follows: (1) studies included healthy children without systemic diseases; (2) in vivo studies involving caries-damaged primary teeth; (3) in vitro studies conducted with either intact or caries-affected primary teeth; (4) microbiological studies using bacteria samples isolated from children’s saliva or dental plaque; (5) studies published between 2017 and 2022, inclusive; (5) articles published in the English language; (6) access to the full-text version of the articles.

Conversely, the criteria for exclusion during the article selection process were as follows: (1) studies involving children with systemic diseases; (2) in vivo studies conducted on permanent teeth in children; (3) in vitro studies using extracted permanent teeth; (4) in vitro studies involving laboratory-cultivated bacteria obtained from biological resource centres; (5) research conducted on animals; (6) articles published prior to 2017; (7) literature reviews, meta-analyses, case reports, conference papers, letters, theses, books, and dissertations. Studies involving children with systemic diseases were excluded to ensure a homogeneous population and minimise confounding variables that could affect treatment outcomes.

### 2.6. Data Extraction

A narrative synthesis was conducted by two independent review authors, K.R. and J.R., using studies that met the inclusion criteria. The pertinent information was synthesised by tabulating the data according to (1) authors, (2) publication year, (3) country of study, (4) study design, (5) sample size, (6) SDF concentration and application technique, (7) methods used to evaluate the mechanisms of action of SDF, (8) outcome measures, (9) study period, and (10) results. Within the tables, the studies were systematically arranged based on their primary findings. This structured presentation encapsulated a diverse range of outcomes, encompassing both quantitative and qualitative shifts in oral microflora, alterations in microhardness of hard tissues, modifications in mineral density and composition within primary teeth, and changes in clinical characteristics associated with the various application methodologies.

### 2.7. Risk of Bias Assessment

The risk of bias in the included studies was assessed by two independent reviewers, E.A.B. and V.A. Two authors (E.A.B. and V.A.) initially analysed the guidelines for using the tools and practised evaluating the risk of bias in a randomly selected sample of five in vivo and five in vitro studies, discussing their findings. Subsequently, E.A.B. and V.A. independently and in duplicate assessed 19 articles for this systematic review to identify any discrepancies. If a consensus could not be reached, discussions were held with the third author, J.N., to resolve these differences. The inter-examiner Kappa score was 0.91.

The methodological quality of in vivo studies was evaluated using the Cochrane Collaboration Risk of Bias Assessment Tool [30]. If the specified criteria are met, the risk of bias is considered low and marked with a ‘+’. If the risk is high, it is marked with a ‘×’. If there are insufficient data to assess the risk, it is marked with a ‘−’. The risk of bias is considered low if all criteria are met, unclear if one criterion is not met or the risk of bias is unclear for two criteria, and high if two or more criteria are not met.

In vitro studies were evaluated using the QUIN Tool (Quality Assessment Tool For In Vitro Studies) [31], which consists of 12 bias criteria. Each criterion is scored from 0 to 2, with 0 indicating ‘not specified’, 1 indicating ‘inadequately specified’, and 2 indicating ‘adequately specified’. If a criterion is not relevant, it is marked as ‘not applicable’ and excluded from the calculations. The maximum study score is 24 points, and studies are classified as low risk (>70% score), moderate risk (50–70% score), or high risk of bias (<50% score).

### 2.8. Statistical Analysis

Due to the considerable heterogeneity among the studies, a meta-analysis was not performed. Instead, the results from the studies are presented using percentage expressions (%) or as mean values along with their corresponding standard deviations, typically represented as X ± Y. The research results were considered statistically significant when the *p*-value is less than 0.05.

## 3. Results

### 3.1. Study Selection

**Figure 1:**
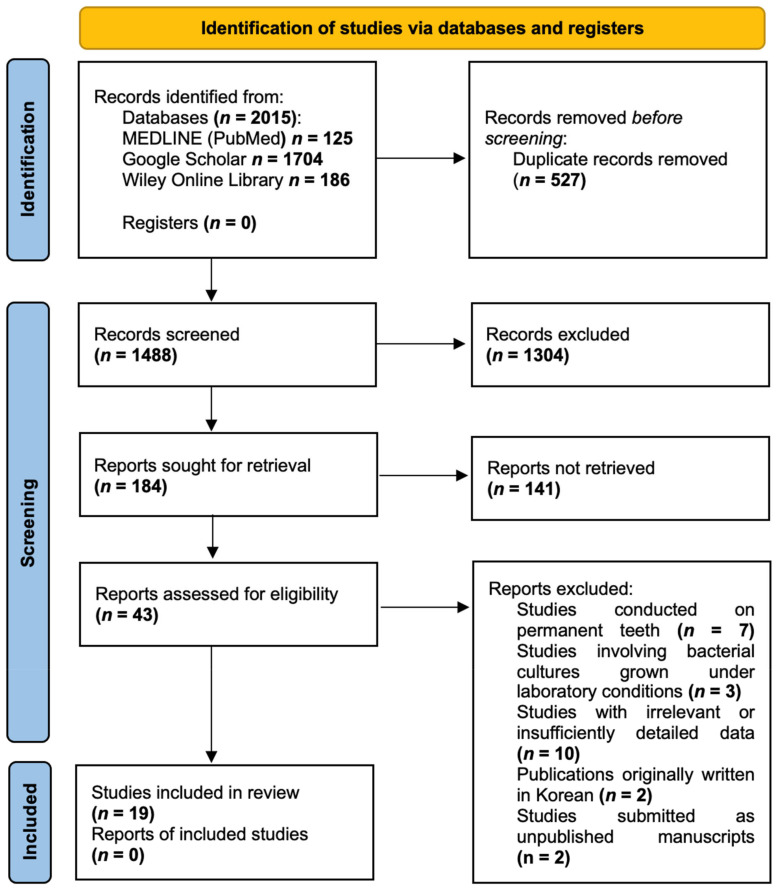
PRISMA Flow diagram.

After conducting the search, a total of 2015 publications were identified in the databases. Duplicate articles (*n* = 527) were removed using the bibliographic information management programme Zotero (Roy Rosenzweig Center for History and New Media, Arlington, TX, USA). Upon screening the remaining 1488 publications and carefully assessing their titles and abstracts, 1445 studies were deemed ineligible for further consideration. Consequently, 43 articles were selected for full-text analysis. After a thorough examination of these full-text articles and strict adherence to the inclusion and exclusion criteria, 19 publications were included in the review. The PRISMA Flow diagram (Figure 1) provides a detailed illustration of the publication selection process. The list of excluded publications is provided in Table S2 (Supplementary Materials).

### 3.2. Quality Assessment

**Figure 2:**
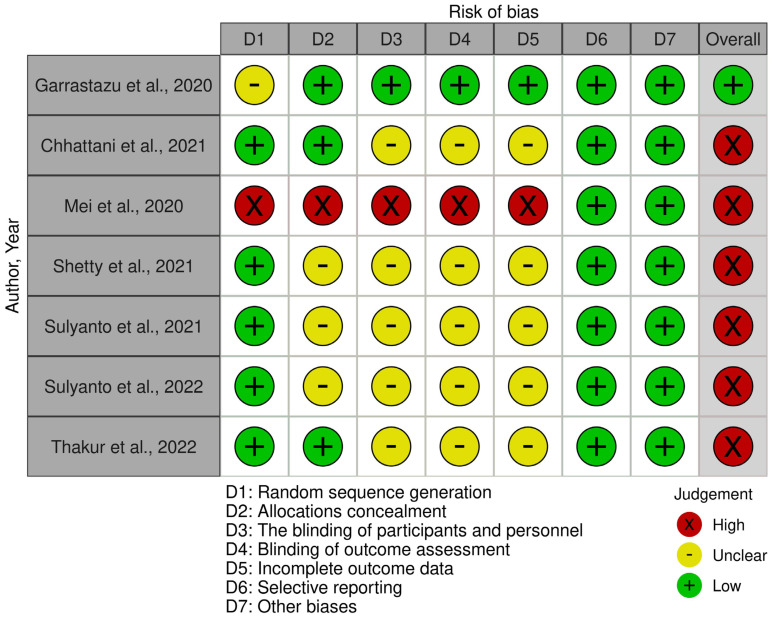
Methodological quality of in vivo studies [32,33,34,35,36,37,38] Garrastazu et al. [32], Chhattani et al. [33], Mei et al. [34], Shetty et al. [35], Sulyanto et al., 2021 [36], Sulyanto et al., 2022 [37], Thakur et al. [38].

Following the evaluation of the methodological quality of in vivo studies, it was found that one study [32] had a low risk, while six studies [33,34,35,36,37,38] had a high risk of bias. Regarding in vitro studies, eight studies [39,40,41,42,43,44,45,46] were determined to have a low risk, while the remaining four studies [47,48,49,50] had a moderate risk of bias. Detailed assessments of the risk of bias in both in vivo and in vitro studies is presented in Figure 2 and Table 2, respectively.

### 3.3. Characteristics of Included Studies

The systematic review included a total of 19 investigations, consisting of 7 in vivo [32,33,34,35,36,37,38] and 12 in vitro studies [39,40,41,42,43,44,45,46,47,48,49,50]. In vivo studies had various study designs, including a randomised controlled trial [38], case–control study [37], and exploratory trial [32]. Among these 19 studies, ten studies [32,33,34,35,36,37,38,40,41,45] focused on deciduous teeth afflicted by carious lesions attributed to bacterial acids, while the remaining nine studies [39,42,43,44,46,47,48,49,50] employed artificial carious lesions induced through pH cycling protocols. Regarding the SDF concentration used, 14 studies [33,34,35,36,37,38,39,40,41,44,45,46,49,50] utilised 38% SDF, four studies [32,43,47,48] employed 30% SDF, and one study [42] used both 30% and 38% SDF solutions. The duration of SDF applications varied, ranging from as short as 30 s [34,42] to as long as 5 min [41]. The research sample consisted of 793 primary teeth, with 281 teeth belonging to the anterior group, 465 teeth in the posterior group, and 47 teeth not classified into any specific group. The analysis of microbiological changes in saliva and plaque involved the data from 263 children, ranging in age from 1 to 12 years. Among the included studies, three [32,33,35] assessed quantitative changes, two [34,41] evaluated qualitative alterations, and one [37] examined both quantitative and qualitative shifts in oral microflora following SDF applications. Five studies [43,44,47,49,50] scrutinised alterations in microhardness, three studies [36,39,46] investigated differences in mineral density and composition of hard tissues, and the remaining five studies [38,40,42,45,48] explored the impact of various SDF application methods on clinical outcomes. The data of interest regarding the included studies are presented in Table 3, Table 4, Table 5, Table 6 and Table 7.

## 4. Discussion

In contemporary dentistry, conventional caries treatment techniques, such as drilling and filling, have been progressively supplanted by minimally invasive approaches that prioritise the management of dental caries through strategies such as the correction of oral microflora, control of risk factors, remineralisation using fluorides, and ongoing long-term monitoring [51]. The antimicrobial attributes of fluorides, coupled with their capacity to facilitate remineralisation, have proven highly effective in thwarting dental caries. However, with the introduction of SDF, researchers developed an interest in exploring its potential for both preventing and treating dental caries [52]. Zhao et al. [53] conducted a study to elucidate the mechanisms through which SDF operates and showcased its ability to combat oral microflora associated with the development of cavities. Additionally, they highlighted its capacity to stimulate the remineralisation of enamel and dentine. The synergistic action of silver and fluoride ions in SDF works by inhibiting enzymes responsible for carbohydrate metabolism, disrupting bacterial cell walls, and impeding bacterial deoxyribonucleic acid replication. When SDF interacts with dental hydroxyapatite, it forms calcium fluoride. Calcium fluoride is soluble in saliva and serves as a reservoir for fluoride ions [54,55]. At low pH, the fluoride ions released from calcium fluoride can react with phosphate and calcium ions, as well as acid phosphates formed in or released from the enamel during a caries challenge, leading to the re-precipitation of these substances in the form of more acid-resistant fluorhydroxyapatite [56,57]. Furthermore, SDF generates insoluble silver chloride and silver phosphate, creating a protective layer that prevents the loss of calcium and phosphorus ions from demineralised enamel or dentine. This process leads to an increase in microhardness [58].

Upon analysing the literature, it appears that the application of SDF leads to substantial quantitative alterations in oral microflora. In a study conducted by Chhattani et al. [33], SDF exhibited the most pronounced reduction in *Streptococcus mutans* and *Lactobacillus* counts when compared to alternative agents such as CHX and NaF. In particular, *Lactobacilli* were highly sensitive to SDF, to the extent that they failed to form a bacterial community after SDF application. *Mutans streptococci* and *Lactobacilli* are well known as cariogenic oral bacteria [59]. Excessive acidification of the oral environment by aciduric species is directly associated with the development of dental caries [60]. Poor oral hygiene and mouth bleeding can facilitate *Streptococcus mutans* entering the bloodstream, potentially leading to systemic diseases. Pathogenic bacterial colonisation is closely linked to inflammation and cancer progression [61]. Poor oral health disrupts the microbiome balance and is associated with dysplasia and carcinogenesis in head and neck cancer [62]. Additionally, oral microflora can cause severe infections, leading to complications such as sepsis, meningitis, and abscesses in the central nervous system [63]. On the other hand, several *Lactobacillus* species, including *Lactobacillus salivarius*, *Lactobacillus fermentum*, and *Lactobacillus paracasei*, have been studied for their potential to combat cariogenic bacteria. However, the idea of *Lactobacilli* being beneficial in the context of dental cariogenesis remains controversial [64]. Thus, a limitation of the Chhattani et al. [33] study is the lack of specification of the types of *Lactobacilli*, making it difficult to accurately assess their influence on the pathogenesis of dental caries. Shetty et al. [35] similarly reported significant antibacterial properties of SDF against *Streptococcus mutans*, resulting in a substantial reduction in colony-forming units after repeated applications. Garrastazu et al. [32] observed a similar effect of SDF and CHX in reducing *Streptococcus mutans* counts in saliva, with a significant decrease at 24 h and 30 days. However, there was an increase at 90 days, possibly influenced by diminishing antimicrobial effectiveness and patient-related factors such as regarding oral hygiene compliance. In contrast, Sulyanto et al. [37] found a lower percentage of live–dead bacteria in SDF-treated plaque compared to SDF-untreated plaque samples, although this difference did not reach statistical significance. The authors emphasised the influence of various factors, including saliva composition, nutrition, and oral hygiene, which can impact the microbial population. Additionally, it is essential to note that this study involved the application of fluoride varnish to primary teeth. Yu et al. [65] observed a detrimental effect of NaF on the antibacterial properties of SDF. The components of fluoride varnish were found to attach to silver ions, diminishing their binding capacity to bacterial cells.

The findings from both in vivo and in vitro studies consistently reveal similar results when assessing changes in the species composition of the oral cavity microflora. Sulyanto et al. [37] did not observe a significant difference in the communities of bacteria forming dental plaque on tooth surfaces affected by caries before and after SDF treatment. However, a statistically significant difference emerged when analysing the dentine of arrested lesions. It is worth mentioning that in subsurface carious dentine treated with silver diamine fluoride, there was an increase in *Lactobacillus rhamnosus*. This species is extensively used as a probiotic in food formulations, health products, and functional foods [66]. After undergoing SDF treatment, there was a notable increase in the abundance of early colonisers such as *Streptococcus salivarius*, *Streptococcus gordonii*, and *Actinomyces odontolyticus*. These microorganisms have an affinity for smooth surfaces and contribute to oral health by producing alkali, bacteriocins, and hydrogen peroxide. Similarly, Liu et al. [41] confirmed a decrease in bacterial diversity after SDF treatment, although this change did not reach statistical significance. The authors observed that complex commensal relationships between different microorganism species became more prominent in dental plaque, accompanied by a significant reduction in carbohydrate transport and metabolic functions. Mei et al. [34] did not identify a statistically significant alteration in microbial diversity between arrested and active caries lesions. However, there was a declining trend in species composition. Furthermore, a limitation of this study was the lack of specification regarding the exact species of *Lactobacillus*. Interestingly, a higher diversity of acid-producing microorganisms was found in active caries lesions after SDF treatment compared to the beginning of the study. These findings align with a review conducted by Zhang et al. [67]. The authors summarised data from 21 investigations on the impact of SDF on oral biofilm. They observed that SDF tends to reduce the number of major caries pathogens such as *Streptococcus mutans* and *Lactobacillus*. However, this agent did not substantially impact all microorganisms within the oral cavity ecosystem. As the population of cariogenic microorganisms decreases, a new ecological balance is established in the oral cavity. *Lactobacillus acidophilus* and *Lactobacillus rhamnosus* are routinely found in both deep and superficial carious lesions [68,69]. *Lactobacillus rhamnosus* is not considered to be cariogenic because it cannot ferment sucrose or lactose [70]. *Lactobacillus acidophilus* has a dual role: it is beneficial in the gut but potentially cariogenic in the mouth. It can significantly increase salivary pH and reduce the levels of *Streptococcus mutans* in saliva. However, the lactic acid produced by *Lactobacillus acidophilus* from carbohydrate fermentation, along with *Streptococcus mutans*, is responsible for the demineralisation of tooth enamel that triggers dental caries [71,72].

The studies conducted by Sai et al. [44] and Abdil-Nafaa and Qasim [47] reported a statistically significant enhancement in the microhardness of primary teeth following the application of SDF. When compared to an experimental formulation containing silver nanoparticles (Ag-Nano), SDF, as investigated by Scarpelli et al. [50], yielded a notable increase in the microhardness of primary teeth. As an alternative to SDF, Reis et al. [43] suggest the use of bioactive giomer varnish. The application of this material resulted in a significant elevation in microhardness compared to applications involving distilled water and 30% SDF solution. However, Mohammadi and Farahmand Far [49] did not find a statistically significant increase in enamel microhardness when using SDF. The authors pointed out that the remineralisation effect of SDF is more effective for dentine caries. In contrast to enamel, dentine contains higher levels of proteins, carbonates, and phosphates, which can react more readily with silver ions. In cases of enamel demineralisation, new remineralising methods alternative to fluoride, based on the integration of calcium and phosphates at the level of demineralised dental surfaces, can be applied, such as biomimetic hydroxyapatite, which reduces the incidence of white spot lesions, hypersensitivity, and the occurrence of caries [73].

When evaluating the effectiveness of SDF in remineralisation, significant alterations in mineral density and the composition of hard tissues have been noted in both in vivo and in vitro studies. Yılmaz et al. [46] observed that SDF resulted in a statistically significant increase in mineral density. The mineral density values reported in their study were 20–30% higher than those found in other studies. This variation might be attributed to the artificial demineralisation lesions created over a short period, which differ in depth compared to lesions caused by the natural caries process in the oral cavity. Additionally, the surface layers contain a higher density of mineral substances. Abdellatif et al. [39] also reported similar outcomes, where SDF exhibited significantly higher remineralisation efficiency when compared to NaF. In the SDF group, the average increase in calcium ions was 48%, and the calcium–phosphorus ion ratio increased by 24%. However, no significant difference was observed between the groups when assessing the percentage changes in phosphorus ions. Sulyanto et al. [36] found that silver ions can penetrate dentine up to a depth of 0.6 mm. While most of the silver particles initially permeate through the dentinal tubules after application, the gradual increase in SDF penetration depth and the occlusion of dentinal tubules over a period of three weeks suggest that these processes are time dependent. The authors also noted the deposition of zinc (Zn) ions in caries-affected dentine and around the pulp chamber. Zn ions are believed to play a crucial role in the biomineralisation of dental tissues. Dentine mineralisation occurs in a spherical manner, with dentine calcospherites merging together. Zn ions are present in these dentine calcospherites, implying their significance in the formation of tertiary dentine. Furthermore, SDF treatment leads to the formation of a thicker tertiary dentine layer in deciduous teeth.

There is a growing interest in exploring various application methods of SDF to achieve the best clinical results. Thakur et al. [38] did not find a statistically significant correlation between the number of arrested dental caries lesions and the duration of SDF application. Similarly, Punhagui et al. [42] investigated changes in enamel microhardness of primary teeth resulting from varying concentrations and durations of SDF application. They did not find any statistically significant links between SDF concentration, application time, and enamel microhardness values. Toopchi et al. [45] observed that modifying the traditional SDF application by adding 40 s of light curing resulted in a 26% increase in the microhardness of infected dentine. Similarly, Hassan et al. [40] reported that surface microhardness values were 2.3 times higher compared to the traditional application technique. Nevertheless, Toopchi et al. [45] noticed that the penetration depth of SDF decreased by two-fold after light polymerisation. It is believed that the emitted light accelerates the photochemical reduction in SDF, resulting in improved penetration when the traditional application method is used. Research has shown that SDF releases, on average, 2–3 times more fluoride ions compared to other fluoride compounds, such as NaF, sodium fluoride monophosphate, and stannous fluoride [74,75]. Deeper penetration of SDF is clinically significant as it ensures more effective remineralisation of caries-damaged hard tissues. Significant clinical results have been achieved with the simultaneous application of SDF and laser ablation. Hassan et al. [40] reported that dentine microhardness increased by 3.3 and 7.6 times after using the erbium, chromium-doped yttrium, scandium, gallium, and garnet laser compared to the light curing and the traditional application technique groups, respectively. A synergistic mechanism of action was observed when SDF was used in conjunction with grape seed extract. Hussein et al. [48] found that dentine microhardness increased by 24% when SDF and grape seed extract were applied simultaneously, compared to the traditional application method. Grape seed extract contains proanthocyanidin, a polyphenol that forms cross-links with intact and caries-damaged dentine, strengthens collagen fibres, and inhibits their breakdown [76]. SDF also inhibits collagen fibre breakdown by regulating the activity of matrix metalloproteinases, which are enzymes with collagenolytic properties [77].

The studies included in this review have several limitations. Due to the challenges associated with working with child patients and concerns regarding the potential impact of SDF on the colour of primary teeth, most of the relevant studies opted to use in vitro methods for their investigations. It is important to acknowledge that in vitro studies provide the lowest level of research evidence. Artificial caries lesions created in laboratory settings using the pH cycle model cannot fully replicate the complex pathological process of dental caries in the oral cavity. Additionally, critical factors contributing to caries development, such as dietary habits, individual oral hygiene, fluoride intake, and the qualitative and quantitative components of saliva, cannot be adequately accounted for in these studies. SDF is recognised as a cost-effective material for the treatment of dental caries, which makes it particularly accessible in economically disadvantaged regions. As a result, the majority of studies included in this review were conducted in countries such as India, Brazil, and several Asian nations. However, the methodological quality of these studies often falls short of ideal standards, thereby increasing the risk of systematic errors and potential biases in the findings. An assessment of the methodological quality revealed that four studies had a moderate risk of bias, while six studies had a high risk of bias. Consequently, the findings presented in this review may be either falsely significant or insignificant. Finally, when assessing the potential of SDF in relation to the study objectives, one predominant study design (either in vivo or in vitro) was observed among the included studies, which limits the comparability of results obtained from laboratory studies to those obtained from clinical trials involving children.

The findings of our study reaffirm the compelling evidence supporting the substantial remineralisation potential of SDF. Notably, SDF exhibits superior efficacy in managing cavitated caries lesions with exposed dentine compared to other remineralising agents, which predominantly target enamel. This conclusion is consistent with the guidelines set forth by the American Academy of Pediatric Dentistry (AAPD), which identifies cavitated lesions as a primary indication for the application of SDF. However, our investigation did not demonstrate statistically significant correlations between SDF concentration, duration of application, and treatment efficacy, thereby constraining our ability to formulate definitive clinical recommendations. The AAPD guidelines advocate for the use of 38% SDF with an optimal application duration of one minute, while acknowledging that the duration of application in clinical studies does not uniformly correlate with treatment outcomes. Furthermore, although the U.S. Food and Drug Administration approved SDF for the reduction of tooth sensitivity in 2014, and the World Health Organisation included it in the core list of essential medicines for the prevention and treatment of dental caries in 2021 [78,79], the evidence supporting its broader application in caries management remains limited. The off-label utilisation of SDF by healthcare professionals underscored its potential benefits, but simultaneously highlights the critical need for more extensive and rigorous clinical trials. Such research is essential to establish a robust scientific framework that could facilitate the expansion of approved indications for SDF, define evidence-based protocols for its application, and ultimately improve dental care practises and patient outcomes. Additionally, exploring the molecular mechanisms by which SDF promotes remineralisation and investigating its long-term effects on dental structures could yield valuable insights that enhance our understanding of its therapeutic potential and optimise its application in clinical settings.

## 5. Conclusions

SDF demonstrates significant efficacy in suppressing the proliferation of microorganisms associated with dental caries, while promoting a slight shift in species composition towards restoring normal oral microflora. Furthermore, SDF increases the mineral density of hard dental tissues, enabling effective remineralisation of carious lesions in both enamel and dentine, which results in a measurable rise in microhardness. The integration of conventional SDF application techniques with advanced modalities, such as light curing or laser ablation, yields a notable improvement in the microhardness of primary teeth.

## Figures and Tables

**Table 1:** PICO search strategy.

Framework Item	Description
Population	Caries-affected (in vivo, in vitro) or intact (in vitro) primary teeth extracted for orthodontic reasons
Intervention	Application of varying concentrations of SDF to caries lesions. In in vitro studies involving intact teeth, artificial caries lesions were created by subjecting the teeth to demineralising and remineralising solutions with varying pH levels
Comparison	The potential of SDF is assessed either without control or in comparison with placebo or other prophylactic agents with antibacterial or remineralising properties, such as NaF, silver nitrate, chlorhexidine digluconate (CHX), etc. The influence of different application methods of SDF on clinical outcomes is also compared
Outcome	The outcomes of interest include the diversity and quantity of oral cavity microflora, changes in mineral density and mineral content of hard tissues, as well as alterations in microhardness of demineralised tissues (enamel or dentine)

**Table 2:** Methodological quality of in vitro studies.

Author, Year	Abdellatif et al., 2022 [39]	Abdil-Nafaa and Qasim, 2020 [47]	Hassan et al., 2021 [40]	Hussein et al., 2021 [48]	Liu et al., 2020 [41]	Mohammadi and Farahmand Far, 2018 [49]	Punhagui et al., 2021 [42]	Reis et al., 2021 [43]	**Sai et al., 2020** [44]	**Scarpelli et al., 2017** [50]	**Toopchi et al., 2021** [45]	**Yılmaz et al., 2020** [46]
Criteria												
Clearly stated aims/objectives	2	2	2	2	2	2	2	2	2	0	2	2
Detailed explanation of sample size calculation	2	0	2	0	0	0	1	2	0	0	2	2
Detailed explanation of sampling technique	2	2	2	2	2	2	2	2	2	2	2	2
Details of comparison group	2	2	2	2	not applicable	2	2	2	2	2	2	2
Detailed explanation of methodology	2	2	2	2	2	2	2	2	2	2	2	2
Operator details	0	0	0	0	0	0	0	0	1	0	0	0
Randomization	2	1	1	1	not applicable	1	2	2	1	1	1	1
Method of measurement of outcome	2	2	2	2	2	2	2	2	2	2	2	2
Outcome assessor details	0	0	0	0	0	0	0	0	1	0	0	0
Blinding	0	0	0	0	not applicable	0	0	0	1	0	0	0
Statistical analysis	2	2	2	2	2	1	2	2	2	2	2	2
Presentation of results	2	2	2	2	2	1	2	1	2	2	2	2

Abbreviations. 0—not specified; 1—inadequately specified; 2—adequately specified.

**Table 3:** Characteristics of studies analysing quantitative changes in microflora of the oral cavity.

Author, Year	Country	Study Design	Sample Size	Concentration of SDF	Time of Sampling	Quantitative Changes in Oral Microflora ± SD		
Chhattani et al., 2021 [33]	India	In vivo	90 3–9-year-old children	38%	Baseline	Blood agar: 400.83 CFU	*Lactobacillus* agar: 150.43 CFU	*Mitis salivarius* agar: 192.2 CFU
					After a 21-day period	Blood agar: 66.3 ± 73.91 CFU	*Lactobacillus* agar: 22.1 ± 31.85 CFU	*Mitis salivarius* agar: 43.83 ± 52.52 CFU
					Baseline	Biofilm formation		
						*Lactobacillus* agar: 0.026 ± 0.005		*Mitis salivarius* agar: 0.024 ± 0.004
					After a 21-day period	*Lactobacillus* agar: 0		*Mitis salivarius* agar: 0.003 ± 0.008
Garrastazu et al., 2020 [32]	Brazil	Exploratory trial	90 6–10-year-old children	30%	Baseline	8.79 × 10^7^ + 4.62 × 10^8^		
					After 24 h	3.92 × 10^6^ + 5.92 × 10^6^		
					After 30 days	3.06 × 10^4^ + 3.09 × 10^4^		
					After 90 days	2.94 × 10^6^ + 5.97 × 10^6^		
Shetty et al., 2021 [35]	India	In vivo	22 3–6-year-old children	38%	Baseline	5.21 ± 0.88 × 10^6^ CFU/mL		
					After 3 days	1.88 ± 0.58 × 10^6^ CFU/mL		
					After 6 months	0.88 ± 0.53 × 10^6^ CFU/mL		
					3 days after the reapplication at 6 months	0.3 ± 0.23 × 10^6^ CFU/mL		
Sulyanto et al., 2022 [37]	USA	Case–control study	13 1–4-year-old children	38%	After 8–12 weeks	Percentage of dead microbes in SDF-treated plaque: 56% Percentage of dead microbes in SDF-untreated plaque: 67%		

Abbreviations. CFU—colony-forming unit, NaF—sodium fluoride, SDF—silver diamine fluoride, SD—standard deviation, USA—United States of America.

**Table 4:** Characteristics of studies analysing qualitative changes in microflora of the oral cavity.

Author, Year	Country	Study Design	Sample Size	SDF, %	Outcome														
Liu et al., 2020 [41]	China	In vitro	5 6–12-year-old children	38%	The most prevalent microorganisms observed in the samples														
					Saliva			Plaque from intact teeth			Plaque from carious teeth			Plaque from SDF-treated teeth after a 24 h period			Plaque from SDF-treated teeth after a 1-week period		
					*Streptococcus*			*Streptococcus*			*Pseudomonas*			*Pseudomonas*			*Pseudomonas*		
					*Neisseria*			*Neisseria*			*Oisenella*			*Streptococcus*			*Oisenella*		
					*Haemophilus*			*Leptotrichia*			*Bifidobacterium*			*Oisenella*			*Bifidobacterium*		
					*Veillonella*			*Actinomyces*			*Streptococcus*			*Veillonella*			*Fusobacterium*		
					*Leptotrichia*			*Veillonella*			*Prevotella*			*Bifidobacterium*			*Pseudoramibacter*		
Mei et al., 2020 [34]	Hong Kong	In vivo	14 5-year-old children	38%	The most prevalent microorganisms observed in the plaque samples from arrested caries lesions														
					Pre-SDF treatment					2 weeks post-SDF treatment					12 weeks post-SDF treatment				
					*Neisseria* sp.					*Neisseria* sp.					*Neisseria* sp.				
					*Leptotrichia* sp.					*Veillonella* sp.					*Leptotrichia* sp.				
					*Veillonella* sp.					*Corynebacterium* sp.					*Veillonella* sp.				
					*Corynebacterium* sp.					*Lauptropia mirabilis*					*Porphyromonas* sp.				
					*Capnocytophaga* sp.					*Capnocytophaga* sp.					*Lauptropia mirabilis*				
					The most prevalent microorganisms observed in the plaque samples from active caries lesions														
					Pre-SDF treatment					2 weeks post-SDF treatment					12 weeks post-SDF treatment				
					*Neisseria* sp.					*Rothia* sp.					*Veillonella* sp.				
					*Leptotrichia* sp.					*Veillonella* sp.					*Neisseria* sp.				
					*Veillonella* sp.					*Streptococcus mutans*					*Leptotrichia* sp.				
					*Corynebacterium* sp.					*Lactobacillus* sp.					*Rothia* sp.				
					*Lauptropia mirabilis*					*Corynebacterium* sp.			*Corynebacterium* sp.						
Sulyanto et al., 2022 [37]	USA	Case–control study	29 1–4-year-old children	38%	Mean abundance of microbial species on intact enamel and carious surface biofilm														
					Intact enamel					Pre-SDF treatment					Post-SDF treatment				
					*Streptococcus mitis*: 10.2%					*Veillonella atypica*: 15.9%					*Rothia dentocariosa*: 16.8%				
					*Veillonella atypica*: 9.3%					*Rothia dentocariosa*: 14.6%					*Veillonella atypica*: 14.2%				
					*Haemophilus parainfluenzae*: 7.8%					*Streptococcus mutans*: 10%					*Streptococcus mitis*: 8.7%				
					*Rothia dentocariosa*: 7.3%					*Streptococcus mitis*: 6.4%					*Streptococcus mutans*: 6.5%				
					*Neisseria flava*: 5.8%					*Prevotella histicola*: 3.3%					*Rothia aeria*: 3.2%				
					Mean abundance of species in subsurface carious dentine														
					Post-SDF treatment					No SDF treatment									
					*Lactobacillus casei rhamnosus*: 10.1%					*Streptococcus mutans*: 28.7%									
					*Veillonella atypica*: 9.3%					*Veillonella atypica*: 9.9%									
					*Actinomyces viscosus*: 8.7%					*Parascardovia denticolens*: 6.2%									
					*Streptococcus mutans*: 8.2%					*Scardovia wiggsiae*: 5.4%									
					*Parascardovia denticolens*: 6.5%					*Actinomyces IP073*: 4.6%									

Abbreviations. SDF—silver diamine fluoride, sp.—species, USA—United States of America.

**Table 5:** Characteristics of studies analysing changes in microhardness of the hard tissues of primary teeth.

Author, Year	Country	Study Design	Sample Size	Concentration of SDF	Assessment of Microhardness	Assessment Time	**Microhardness Values ± SD**
Abdil-Nafaa and Qasim, 2020 [47]	Iraq	In vitro	150 anterior primary teeth	30%	Vickers microhardness tester (OTTO Wolpert–WERKE GMBH, Ludwigshafen, Germany), load of 5 g, time of 15 s	Baseline	204.764 ± 9.36 kgf/mm^2^
						After SDF application and demineralisation cycle	189.882 ± 8.897 kgf/mm^2^
Mohammadi and Farahmand Far, 2018 [49]	Iran	In vitro	45 anterior primary teeth	38%	Microhardness tester Shimadzu HMV-2000 (Shimadzu Corporation, Kyoto, Japan), load of 25 g, time of 5 s	Baseline	252 kgf/mm^2^
						After demineralisation cycle	155 kgf/mm^2^
Reis et al., 2021 [43]	Brazil	In vitro	36 primary teeth	30%	HVS-1000 microhardness tester (Pantec, São Paulo, SP, Brazil), load of 5 g, time of 5 s	After demineralisation cycle	36.1 ± 9.95 kgf/mm^2^
						30 days post-SDF application	39.3 ± 7.31 kgf/mm^2^
Sai et al., 2020 [44]	India	In vitro	30 anterior primary teeth	38%	Digital Micro Vickers hardness tester, load of 200 g, time of 20 s	Baseline	300.58 ± 27.58 kgf/mm^2^
						After demineralisation cycle	244.76 ± 25.28 kgf/mm^2^
						2 weeks post-SDF application	394.25 ± 47.66 kgf/mm^2^
Scarpelli et al., 2017 [50]	Brazil	In vitro	100 primary molars	38%	Knoop-type penetrator (HMV-G; Shimadzu, Tokyo, Japan), load of 25 g, time of 5 s	8 days post-SDF application	28.55 ± 11.75%

Abbreviations. SD—standard deviation, SDF—silver diamine fluoride.

**Table 6:** Characteristics of studies examining changes in mineral density and mineral composition of the hard tissues of primary teeth.

Author, Year	Country	Study Design	Sample Size	Concentration of SDF	Technique for Assessment	Outcome	
Abdellatif et al., 2022 [39]	Egypt	In vitro	40 anterior primary teeth	38%	Energy dispersive X-ray spectroscopy	Ca content in dentine ± SD	
						Baseline	24.86 ± 1.55%
						After demineralisation cycle	18.54 ± 2.34%
						After remineralisation cycle	28.69 ± 2.26%
						P content in dentine ± SD	
						Baseline	12.92 ± 0.91%
						After demineralisation cycle	10.7 ± 1.27%
						After remineralisation cycle	13.44 ± 1.62%
						Ca/P ratio in dentine ± SD	
						Baseline	1.92 ± 0.05%
						After demineralisation cycle	1.73 ± 0.1%
						After remineralisation cycle	2.14 ± 0.16%
Sulyanto et al., 2021 [36]	USA	In vivo	11 primary teeth	38%	X-ray fluorescence, energy-dispersive X-ray spectroscopy, microcomputed tomography	SDF penetration depth in SDF-minutes group	~0.5 ± 0.02 mm
						SDF penetration depth SDF-weeks group	~0.6 ± 0.05 mm
						The number of dentinal tubules occluded with Ag ions in SDF-minutes group	6%
						The number of dentinal tubules occluded with Ag ions in SDF-weeks group	20%
						The highest counts of Zn ions	Carious dentine, around the pulp chamber
						The lowest counts of Zn ions	Sound dentine, inside the pulp chamber
Yılmaz et al., 2020 [46]	Turkey	In vitro	54 primary molars	38%	Micro-computed tomography	Mineral density value ± SD	
						Baseline	1.376 ± 0.07 gHApcm^−3^
						After demineralisation cycle	0.961 ± 0.221 gHApcm^−3^
						After remineralisation cycle	1.623 ± 0.171 gHApcm^−3^

Abbreviations. Ag—silver, Ca—calcium, Ca/P—calcium and phosphorus, SD—standard deviation, SDF—silver diamine fluoride, P—phosphorus, USA—United States of America, Zn—zinc.

**Table 7:** Characteristics of studies analysing the impact of SDF application methods on clinical outcomes.

Author, Year	Country	Study Design	Sample Size	Outcome Measures	Follow-Up	SDF Application Technique	**Results**	
Hassan et al., 2021 [40]	Saudi Arabia	In vitro	30 primary molars	Superficial microhardness ± SD	After 1 month	38% SDF 30 s + Er, Cr:YSGG laser 10 s	891.24 ± 37.33 kgf/mm^2^	
						38% SDF 30 s + 40 s light curing	266.65 ± 90.81 kgf/mm^2^	
						38% SDF 30 s	117.91 ± 19.19 kgf/mm^2^	
Hussein et al., 2021 [48]	Egypt	In vitro	60 primary second molars	Percentage change in dentine microhardness ± SD	After 1 week	30% SDF 3 min	60.96 ± 9.89%	
						6.5% grape seed extract 10 min + 30% SDF 3 min	85.03 ± 5.52%	
Punhagui et al., 2021 [42]	Brazil	In vitro	45 primary molars	Percentage of surface remineralisation ± SD	48 h after SDF application	30% SDF 1 min	30.82 ± 13.60%	
						30% SDF 3 min	34.49 ± 13.67%	
						38% SDF 1 min	31.47 ± 18.77%	
						38% SDF 3 min	28.54 ± 8.59%	
Thakur et al., 2022 [38]	India	Randomised controlled trial	176 primary molars 16 primary incisors	Percentage of arrested caries lesions	After 3 weeks	38% SDF 30 s	73.33%	
						38% SDF 1 min	72.29%	
						38% SDF 2 min	86.92%	
					After 3 months	38% SDF 30 s	74.31%	
						38% SDF 1 min	75.86%	
						38% SDF 2 min	82.45%	
					After 6 months	38% SDF 30 s	79.15%	
						38% SDF 1 min	77.29%	
						38% SDF 2 min	75.96%	
Toopchi et al., 2021 [45]	Saudi Arabia	Ex vivo	16 primary incisors	SDF penetration depth ± SD	After 1 month	38% SDF 1 min	130 ± 50 μm	
						38% SDF 1 min + 40 s light curing	60 ± 10 μm	
				Superficial microhardness ± SD		38% SDF 1 min	558.07 ± 119.08 kgf/mm^2^	
						38% SDF 1 min + 40 s light curing	702.26 ± 144.6 kgf/mm^2^	
				Silver ion precipitation ± SD		38% SDF 1 min	Infected dentine	9.28 ± 3.53%
							Affected dentine	5.22 ± 5.07%
							Sound dentine	1.56 ± 1.96%
						38% SDF 1 min + 40 s light curing	Infected dentine	24.61 ± 14.74%
							Affected dentine	4.51 ± 4.15%
							Sound dentine	1.19 ± 1.28%
				Fluoride ion precipitation ± SD		38% SDF 1 min	Infected dentine	0.231 ± 0.19%
							Affected dentine	0.289 ± 0.31%
							Sound dentine	0.34 ± 0.37%
						38% SDF 1 min + 40 s curing	Infected dentine	0.259 ± 0.63%
							Affected dentine	0.38 ± 0.42%
							Sound dentine	0.452 ± 0.51%

Abbreviations. Er, Cr:YSGG—erbium, chromium-doped yttrium, scandium, gallium, and garnet, SD—standard deviation, SDF—silver diamine fluoride.

## Data Availability

Data are contained within the article.

## Supplementary Materials

The following supporting information can be downloaded at https://www.mdpi.com/article/10.3390/medicina60111738/s1: Table S1: Search strategy; Table S2: List of excluded articles [27,79,80,81,82,83,84,85,86,87,88,89,90,91,92,93,94,95,96,97,98,99,100,101,102].
