# Supplementary material for: The Potential of Silver Diamine Fluoride in Non-Operative Management of Dental Caries in Primary Teeth: A Systematic Review
Source: Medicina (Kaunas). 2024 Oct 23;60(11):1738. doi: 10.3390/medicina60111738 (PMC11596966; doi:10.3390/medicina60111738)
Supplement: Supplementary file 1 [file medicina-60-01738-s001.zip › Supplementary Materials S1.pdf]

medicina

MDPI

Table S1. Search strategy.

Search date	Database	Keywords	Results
**2022-10-16**	Google Scholar	("silver diamine fluoride" OR "silver diammine fluoride") AND ("dental plaque" OR ("dental" AND "plaque") OR "oral biofilm" OR ("oral" AND "biofilm") OR "antibacterial activity" OR ("antibacterial" AND "activity") OR "antibacterial effect" OR ("antibacterial" AND "effect") OR "antibacterial potential" OR ("antibacterial" AND "potential") OR "antibacterial efficacy" OR ("antibacterial" AND "efficacy") OR "antimicrobial effect" OR ("antimicrobial" AND "effect") OR "antimicrobial activity" OR ("antimicrobial" AND "activity") OR "antimicrobial potential" OR ("antimicrobial" AND "potential") OR "antimicrobial efficacy" OR ("antimicrobial" AND "efficacy") OR "microbiota")	701
**2022-10-16**	Google Scholar	("silver diamine fluoride" OR "silver diammine fluoride") AND ("dentin remineralization" OR ("dentin" AND "remineralization") OR "enamel remineralization" OR ("enamel" AND "remineralization”) OR "microhardness" OR "dentin demineralization" OR ("dentin" AND "demineralization") OR "enamel demineralization" OR ("enamel" AND "demineralization"))	1003
**2022-10-16**	MEDLINE (PubMed)	("silver diamine fluoride"[All Fields] OR "silver diammine fluoride"[All Fields]) AND ("dental plaque"[All Fields] OR "dental plaque"[MeSH Terms] OR ("dental"[All Fields] AND "plaque"[All Fields]) OR "oral biofilm"[All Fields] OR ("oral"[All Fields] AND "biofilm"[All Fields]) OR "antibacterial activity"[All Fields] OR ("antibacterial"[All Fields] AND "activity"[All Fields]) OR "antibacterial effect"[All Fields] OR ("antibacterial"[All Fields] AND "effect"[All Fields]) OR "antibacterial potential"[All Fields] OR ("antibacterial"[All Fields] AND "potential"[All Fields]) OR "antimicrobial effect"[All Fields] OR ("antimicrobial"[All Fields] AND "effect"[All Fields]) OR "antimicrobial activity"[All Fields] OR ("antimicrobial"[All Fields] AND "activity"[All Fields]) OR "antimicrobial potential"[All Fields] OR ("antimicrobial"[All Fields] AND "potential"[All Fields]) OR "microbiota"[All Fields] OR "microbiota"[MeSH Terms])	60
**2022-10-16**	MEDLINE (PubMed)	("silver diamine fluoride"[All Fields] OR "silver diammine fluoride"[All Fields]) AND ("dentin remineralization"[All Fields] OR ("dentin"[All Fields] AND "remineralization"[All Fields]) OR "enamel remineralization"[All Fields] OR ("enamel"[All Fields] AND "remineralization"[All Fields]) OR "microhardness"[All Fields] OR "dentin demineralization"[All Fields] OR ("dentin"[All Fields] AND "demineralization"[All Fields]) OR "enamel demineralization"[All Fields] OR ("enamel"[All Fields] AND "demineralization"[All Fields]))	65
**2022-10-16**	Wiley Online Library	("silver diamine fluoride" OR "silver diammine fluoride") AND ("dental plaque" OR ("dental" AND "plaque") OR "oral biofilm" OR ("oral" AND "biofilm") OR "antibacterial activity" OR ("antibacterial"	116

		AND "activity") OR "antibacterial effect" OR ("antibacterial" AND "effect") OR "antibacterial potential" OR ("antibacterial" AND "potential") OR "antibacterial efficacy" OR ("antibacterial" AND "efficacy") OR "antimicrobial effect" OR ("antimicrobial" AND "effect") OR "antimicrobial activity" OR ("antimicrobial" AND "activity") OR "antimicrobial potential" OR ("antimicrobial" AND "potential") OR "antimicrobial efficacy" OR ("antimicrobial" AND "efficacy") OR "microbiota")	
**2022-10-16**	Wiley Online Library	("silver diamine fluoride" OR "silver diammine fluoride") AND ("dentin remineralization" OR ("dentin" AND "remineralization") OR "enamel remineralization" OR ("enamel" AND "remineralization”) OR "microhardness" OR "dentin demineralization" OR ("dentin" AND "demineralization") OR "enamel demineralization" OR ("enamel" AND "demineralization"))	70
