# Supplementary material for: The Potential of Silver Diamine Fluoride in Non-Operative Management of Dental Caries in Primary Teeth: A Systematic Review
Source: Medicina (Kaunas). 2024 Oct 23;60(11):1738. doi: 10.3390/medicina60111738 (PMC11596966; doi:10.3390/medicina60111738)
Supplement: Supplementary file 1 [file medicina-60-01738-s001.zip › Supplementary Materials S2.pdf]

medicina

MDPI

Table S2. List of excluded articles.

No.	Author, year	Reason for exclusion
1	Saad et al., 2020	Permanent teeth
2	Soekanto et al., 2018	Permanent teeth
3	Soekanto et al., 2018	Permanent teeth
4	Yu et al., 2018	Permanent teeth
5	Satyanegara et al., 2017	Permanent teeth
6	Srisomboon et al., 2022	Permanent teeth
7	Karched et al., 2019	Permanent teeth
8	Favaro et al., 2022	Bacterial cultures grown under laboratory conditions
9	Moca et al., 2020	Bacterial cultures grown under laboratory conditions
10	Chanratchakool et al., 2022	Bacterial cultures grown under laboratory conditions
11	Romão et al., 2020	Irrelevant or insufficiently detailed data
12	Kunte et al., 2021	Irrelevant or insufficiently detailed data
13	Abdil-Nafaa and Qasim, 2020	Irrelevant or insufficiently detailed data
14	Li et al., 2019	Irrelevant or insufficiently detailed data
15	Thomas et al., 2021	Irrelevant or insufficiently detailed data
16	Milgrom et al., 2018	Irrelevant or insufficiently detailed data
17	Jabin et al., 2021	Irrelevant or insufficiently detailed data
18	Ruff et al., 2021	Irrelevant or insufficiently detailed data
19	Paul et al., 2021	Irrelevant or insufficiently detailed data
20	Piovesan et al., 2021	Irrelevant or insufficiently detailed data
21	Lee et al., 2021	Korean language
22	Seo et al., 2020	Korean language
23	Pornprasertsuk-Damrongsri et al., 2022	Unpublished data
24	Seto et al., 2017	Unpublished data

References

1. Saad AA, Kamel MA, Hamza NK, Niazi HA. The Effect of Silver Diamine Fluoride on Surface Characterization of Demineralized Dentin. Ain Shams Dent J 2020;19:21–8. doi: 10.21608/asdj.2020.140011.
2. Soekanto SA, Rosithahakiki N, Suniarti DF, Sahlan M. Comparison of the potency of several fluoride-based varnishes as an anticariogenic on calcium, phosphate, and fluoride ion levels. Int J Appl Pharm 2018:55–9. doi: 10.22159/ijap.2017.v9s2.14.
3. Soekanto SA, Fadillah F, Nuraisiya P, Gultom F, Sarwono AT. The potential of several fluoride-based varnishes as remineralization agents: morphological studies, dentin surface hardness, and crystallinity tests. Int J Appl Pharm 2018;9:60. doi: 10.22159/ijap.2017.v9s2.15.
4. Yu OY, Mei ML, Zhao IS, Li Q-L, Lo EC-M, Chu C-H. Remineralisation of enamel with silver diamine fluoride and sodium fluoride. Dent Mater Off Publ Acad Dent Mater 2018;34:e344–52. doi: 10.1016/j.dental.2018.10.007. PMID: 30482611.
5. Satyanegara A, Darwita RR, Setiawati F, Adiatman M, Muhammad R. An in vitro study of caries arresting effect of propolis fluoride and silver diamine fluoride on dentine carious lesions. J Int Dent Med Res 2017;10:751–6.
6. Srisomboon S, Kettratad M, Stray A, Pakawanit P, Rojviriya C, Patntirapong S, et al. Effects of Silver Diamine Nitrate and Silver Diamine Fluoride on Dentin Remineralization and Cytotoxicity to Dental Pulp Cells: An In Vitro Study. J Funct Biomater 2022;13:16. doi: 10.3390/jfb13010016. PMID: 35225979.
7. Karched M, Ali D, Ngo H. In vivo antimicrobial activity of silver diammine fluoride on carious lesions in dentin. J Oral Sci 2019;61:19–24. doi: 10.2334/josnusd.17-0366. PMID: 30726799.
8. Favaro JC, Detomini TR, Maia LP, Poli RC, Guiraldo RD, Lopes MB, et al. Anticaries Agent Based on Silver Nanoparticles and Fluoride: Characterization and Biological and Remineralizing Effects – An In Vitro Study. Int J Dent 2022;2022:9483589. doi: 10.1155/2022/9483589. PMID: 35497178.
9. Moca AE, Pelea DC, Negruţiu BM, Burta OL, Vaida LL, Haliţchi G. In-vitro assessment of silver diamine fluoride's effect on a dental plaque specimen. Int J Med Dent 2020;24.
10. Chanratchakool N, Chavengvorakul W, Taweesedt S, Trairatvorakul C, Thanyasrisung P. Effect of Light on the Antibacterial Property of Silver Diamine Fluoride. Srinakharinwirot Univ Dent J 2022;15:86–92.
11. Romão DA, Fernández CE, de Melo Santos L. Commercial Silver Diamine Fluoride (SDF) Products on Caries Lesion Progression in Primary Enamel: An In Vitro Study. Oral Health Prev Dent 2020;18:1024–9. doi 10.3290/j.ohpd.b871057. PMID: 33499554.
12. Kunte S, Gholap C, Deo P, Jagtap C, Desai S, Lakade L. Evaluation of the Histology of Carious Human Primary Incisor after Treatment with 38% Silver Diamine Fluoride. Int J Sci STUDY 2021;9:121–4.
13. Abdil-Nafaa SA, Qasim AA. The Effect of Silver Diamine Fluoride and Fluoride Varnish on Roughness of Primary Teeth Enamel (An In Vitro Study). Al-Rafidain Dent J 2020;20:296–307. doi: 10.33899/rden.2020.127479.1040.
14. Li Y, Liu Y, Psoter WJ, Nguyen OM, Bromage TG, Walters MA, et al. Assessment of the silver penetration and distribution in carious lesions of deciduous teeth treated with silver diamine fluoride. Caries Res 2019;53:431–40. doi: 10.1159/000496210. PMID: 30808824.
15. Thomas CS, Sharma DS, Sheet D, Mukhopadhyay A, Sharma S. Cross-sectional visual comparison of remineralization efficacy of various agents on early smooth surface caries of primary teeth with swept source optical coherence tomography. J Oral Biol Craniofacial Res 2021;11:628–37. doi: 10.1016/j.jobcr.2021.09.006. PMID: 34603951.

16. Milgrom P, Horst JA, Ludwig S, Rothen M, Chaffee BW, Lyalina S, et al. Topical silver diamine fluoride for dental caries arrest in preschool children: A randomized controlled trial and microbiological analysis of caries associated microbes and resistance gene expression. J Dent 2018;68:72–8. doi: 10.1016/j.jdent.2017.08.015. PMID: 28866468.
17. Jabin Z, Nasim I, Vishnu Priya V, Agarwal N. Quantitative Analysis and Effect of SDF, APF, NaF on Demineralized Human Primary Enamel Using SEM, XRD, and FTIR. Int J Clin Pediatr Dent 2021;14:537–41. doi: 10.5005/jp-journals-10005-1988. PMID: 34824510.
18. Ruff RR, Paul B, Sierra MA, Xu F, Li X, Crystal YO, et al. Predicting Treatment Nonresponse in Hispanic/Latino Children Receiving Silver Diamine Fluoride for Caries Arrest: A Pilot Study Using Machine Learning. Front Oral Health 2021;2:695759. doi: 10.3389/froh.2021.695759. PMID: 35048036.
19. Paul B, Sierra MA, Xu F, Crystal YO, Li X, Saxena D, et al. Microbial population shift and metabolic characterization of silver diamine fluoride treatment failure on dental caries. PloS One 2021;16:e0242396. doi: 10.1371/journal.pone.0242396. PMID: 33720954.
20. Piovesan ÉT, Silva MV, de Campos TA, Martins V deP, Bezzerra ACB. Antimicrobial effects of silver diamine fluoride: An in vivo study. Am J Dent 2021;34:49–53. PMID: 33544989.
21. Lee K, Ahn J, Kim JS, Han M, Lee J, Shin J. Effect of Sodium Fluoride Varnish and Potassium Iodide on Remineralization Efficacy of Silver Diamine Fluoride. J Korean Acad Pediatr Dent 2021;48:467–75. doi: 10.5933/jkapd.2021.48.4.467.
22. Seo M, Song J-S, Shin TJ, Hyun H-K, Kim J-W, Jang K-T, et al. The Effect of Silver Diamine Fluoride on Salivary Biofilm. J Korean Acad Pediatr Dent 2020;47:406–15. doi: 10.5933/jkapd.2020.47.4.406.
23. Pornprasertsuk-Damrongsri S, Karnowakul J, Punyanirun K, Jirakran K, Thanyasrisung P, Techatharatip O, et al. Enhanced effectiveness of silver diamine fluoride application with light curing on natural dentin carious lesions: an in-vitro study. Preprint at https://assets.researchsquare.com/files/rs-1377215/v1/8895ad07-b4c1-49e9-97b8-de336775ed57.pdf?c=1646993667 (2022).
24. Seto J, Horst JA, Parkinson DY, Frachella JC, DeRisi JL. Silver microwires from treating tooth decay with silver diamine fluoride. Preprint at https://www.biorxiv.org/content/10.1101/152199v2.full.pdf+html (2017).
